# The conditional effect of family resilience on family quality of life during the Covid-19 pandemic

**DOI:** 10.12688/f1000research.125852.3

**Published:** 2023-01-12

**Authors:** Tery Setiawan, Ria Wardani, Ellen Theresia

**Affiliations:** 1 Faculty of Psychology, Universitas Kristen Maranatha, Bandung, Indonesia; 2 Department of Anthropology and Development Studies, Radboud University Nijmegen, Nijmegen, The Netherlands

**Keywords:** family quality of life (FQOL), family resilience, Covid-19, parental stress, Indonesia

## Abstract

**Introduction**: The Covid-19 pandemic has brought unprecedented challenges, both economically and psychologically, to most families across the world. Yet, little study has been done on this topic in Indonesia, even after the second year of the pandemic. This study examines how the Covid-19 economic impact and parental stress are related to the family quality of life (FQOL) and how the relation is moderated by family resilience.

**Methods**: To this end, we employed previously tested measures to assess the level of parental stress, family resilience and FQOL. Especially for the latter, we modified the measure, developed by Beach Center on Disability, by including only four domains (i.e., family interaction, parenting, emotional well-being, and material well-being) to adjust to our research context.

**Results**: Based on 169 participants, our confirmatory factor analysis (CFA) displays that all employed measures in the study are valid and reliable. Our regression analysis shows that there are significant direct relations of parental stress & family resilience with FQOL. However, we find that family resilience only positively moderates the relation between the Covid-19 economic impact and FQOL but not the relation between parental stress and FOQL; indicating that high family resilience can only buffer the effect of economic impact on FQOL.

**Discussion**: This study presents a view on how the Covid-19 pandemic affects the way families live and hence, their quality of life. In addition, the findings suggest the importance of family relationship and support in times of crisis, not limited to the Covid-19 pandemic.

## Introduction

The emergence of coronavirus disease (Covid)-19 in Indonesia, as in the rest of the world, has changed almost all aspects of human life rapidly. Due to the Covid-19 pandemic, the government implemented a large-scale social restriction policy that involved the closing of offices, factories, and schools as well as putting in place social distance in individuals’ interaction ( Meiliana, 2020). As a result, the policy has forced family members to spend as much time as possible at home, making it more as home confinement ( Wang *et al*., 2020). Children are no longer able to carry out learning activities at school, as well as leisure activities with their peers. In the meantime, parents are also the subject to home confinement. They need to work from home while, at the same time, are expected to oversee their children study at home ( Griffith, 2020).

Although the experience of working from home is likely to be unpleasant for most families, this appears to be a better option than being unemployed or forced to close a business due to the pandemic. Data from the Ministry of Manpower on 27 May 2020 shows that there are at least 1.79 million workers who must be laid off due to the Covid-19 pandemic ( Idhom, 2020). This number is predicted to continue to grow up to four to five million people ( Gusman, 2020). To further complicate, Covid-19 places the elders as individuals with the highest risk of getting infected ( Aronson, 2020). Therefore, the tradition of asking grandparents for help in supervising the children is no longer an option. With health protocols in force, this also means limited opportunity in receiving social support from family, neighbours, and friends. The lack of social support places a greater stress among parents. Based on all this, we argue that the combination of economic hardship and parental stress is likely to have negative impact on family processes, which can be observed in their quality of life ( Hall & Clare, 2003; Hsiao, 2018).

Investigating family quality of life, rather than individual, during the pandemic is important, yet remains little studied; let alone studies in the Asian context, such as Indonesia. In a mundane life, children and parents are an integral part of a society which generally reflect a society’s quality of life (for further explanation, see the ecological system of a family by Bronfenbrenner, 1986). Children’s living condition is heavily contingent on their parents’ living condition, similar to how parents’ satisfaction with their life is largely dependent on their working condition as well as how their children feel and perceive towards their life ( Prime *et al.*, 2020; Thomas *et al.*, 2017). During the Covid-19 pandemic, this claim has become even more evident. A significant change, *e.g.*, unemployment, that occurs within a family is likely to be experienced by the whole system. Therefore, investigating family quality of life (from here onwards is abbreviated FQOL) is of great importance to unfold the potential effects brought by the pandemic.

In relation to the Covid-19 pandemic, parents are deemed to have a certain level of stress in their parental role ( Griffith, 2020). The stress is assumed to be more increased when there are additional demands arise from the pandemic, *e.g.*, uncertainty in employment, medical conditions caused by Covid-19 infection, *etc.* In addition, the response policy to the Covid-19 pandemic may significantly pose external demands to parents, *e.g.*, inability to have quality time outside home. Consequently, parents have a higher chance to run out of psychological resources to be able to regulate their emotions which can affect their effectiveness in their parental role ( Östberg, 1998). As such, the pandemic not only decreases both material and psychological resources among parents, it also heightens their daily stress level. As a result, the heightened stress level may impair their family quality of life ( Hall & Clare, 2003; Hsiao, 2018; Melberg, 2012).

Nevertheless, previous studies have shown that despite economic and psychological obstacles there are some who demonstrate competent family functioning ( Patterson, 2002). According to Walsh (2003), the family’s ability to fulfil their functions of providing basic needs, nurturing children, and other family functions amid ongoing economic and psychological strains is known as family resilience. There are three key family processes that have been shown to protect and even increase family quality of life, namely family belief systems, family organizational patterns, and communication/problem solving processes. All three key processes are argued to reduce stress among family members during the pandemic, ( Prime *et al*., 2020). Therefore, despite economic hardships during the pandemic families with high level of family resilience are expected to be able to deal with the adversity positively and hence, maintain their quality of life.

Taken together, it is of great importance to scrutinize family quality of life using relevant predictors during the Covid-19 pandemic. Yet, as previously mentioned, there is still very little research on the economic impact of Covid-19 on families in Indonesia. When such study exists, either it relies on secondary data ( *cf* Radhitya *et al*., 2020) or it mainly focuses on the economic indicators of the family. Thus, our study has two-fold relevance. First, for a theoretical purpose, we empirically test a theoretical model of risk and protective factors to family quality of life. As such, we are able to delineate and predict to what extent family quality of life is affected by economic aspect of the Covid-19 pandemic. Second, for a methodological purpose, we extend the applicability of FQOL measure in the general family context (see Zuna *et al*., 2009a for a validation of FQOL measure in families without disabilities). In summary, we aim to investigate *to what extent family resilience moderates the relation between Covid-19 economic impact & parental stress during Covid-19 and family quality of life in several cities of Indonesia.* We will do so by employing general population of adults aged 18 above who are married and have, at least, one child.

## Theoretical framework and hypotheses

In this section, we provide brief theoretical explanations related to psychological constructs employed in the study. We carefully selected these constructs based on the literature available. We will start the explanation from the outcome variable and continue to relevant predictors.

### Family quality of life

Historically, quality of life is an interest of scholars in the field of health science, especially patients with disabilities ( Hoffman *et al*., 2006; Hu *et al*., 2011). Its importance has inspired many studies not to focus merely on individuals, but also on family ( *e.g.*, FQOL measure). In 1998, WHO initiated a research attempted to devise a universal instrument of quality of life, which can cover both ‘ill’ and ‘well’ samples ( Division of Mental Health and Prevention of Substance Abuse, 1998). Its exploratory study consisted of patients with ill disease, ‘healthy’ patients, and health care providers. The final study involved 13 countries and 8,294 participants. The validity analyses suggested the measure to include six domains and 24 specific facets, including negative and positive feelings that usually refer to well-being measure. The measure has a two-fold indication. One, quality of life measure is a concern of every individual, regardless of their physical background. Two, the notion greatly overlaps with the concept of well-being; measuring one inevitably includes the other and vice versa. In times of the pandemic, people and their families are likely to be affected by ever-changing and pervasive health policies. Therefore, it is vital to investigate FQOL to predict the impact the pandemic has on a family and protect them against the risk factors resulted from the pandemic.

The conceptualization of FQOL relies on the following theoretical frameworks, (1) systemic concepts, (2) performance concepts, (3) individual-member concepts, and (4) family-unit concepts ( Zuna *et al*., 2009b). In a nutshell, the organizing frameworks suggest that the macro-level systems outside the family, *e.g.*, healthcare, education, along with their policies and programs are associated with individuals’ demographic and characteristics, which directly impact individuals ( Hoffman *et al*., 2006). As each family member is associated to each other and to their habitat, any change experienced by a family member directly (and indirectly) impacts the whole family system ( Samuel *et al*., 2012). Therefore, a substantial change to normality brought by the Covid-19 pandemic is considered to highly impact family quality of life.

Besides health effects, the most direct impact of the Covid-19 pandemic comes from the economic aspect. Due to Covid-19, a lot of business are forced due to a wide-spread lockdown measure. Especially Indonesia, this has led to millions of job loss ( Idhom, 2020). Although the government has provided subsidies for those who lost jobs during the pandemic, the support is not sufficient to cover monthly bills and necessities for the whole family ( Saptoyo, 2021). Based on the family systems theory, the significant economic change will likely to disrupt the family balance ( Samuel *et al*., 2012). The relationship between parents’ adversity and child’s well-being work in a mutually reinforcing system; stress and disruption experienced by one party will affect the other. This significant economic change is expected to impact all dimensions of FQOL ( Hall & Clare, 2003).

In detail, FQOL is generally thought to include dimensions such as closeness, family interaction, family’s financial situation and a room for personal growth ( Hu *et al*., 2011). Based on its conceptualization, most of the FQOL scales emerge as an alternative and practical measure to the use of multiple measures. In this study, we specifically use the Beach Center FQOL scale ( Beach Center on Disability, 2012). The measure consists of five domains, namely family interaction, parenting, emotional well-being, material well-being, and disability-related support. For the purpose of the study that focuses on general population, we retain four dimensions and leave out the last dimension. This use can be equally compared to FQOL scale proposed by Zuna *et al*. (2009a).

### Covid-19 economic impact

Studies show that through aerosol droplets, a single-stranded ribonucleic acid (RNA) of the Covid-19 can infect up to 2.5 noninfected individuals ( Kaye *et al*., 2021). This infection has been shown to cost a human’s life or, at a less severe level, hospitalization. Due to the severity of the disease, the Covid-19 called for a special set of social and health protocols. As a result, the protocols inevitably started to disrupt supply chain of many trades, caused sudden unemployment, and forced closure of a great number of business ( Akbulaev *et al*., 2020; International Labour Organization, 2020). Not only does it pose economic cost on individuals, it has also precipitated many psychosocial impacts such as depression, substance abuse, etc ( Bu *et al.*, 2020; Maital & Barzani, 2020). The latter impact, of course, can also be caused by the former due to a sudden change.

Specifically in Indonesia, the arrival of the Covid-19 has brought many unprecedented challenges to the families. According to a joint survey conducted by UNDP, UNICEF, Prospera and the SMERU Research Instittute (2021), 74.3% of all the households they interviewed during October to November, 2020 admit that their family was earning less than they were in January of the same year. This, in turn, heightened the proportion of low-income households across the country. In specific, this economic impact was felt greater among families in urban areas. The drop in income was also reported in all variety of income groups. On the other hand, the economic impact was exacerbated by increased cost of daily life, such as groceries. Covid-related expenditures, *e.g.*, face mask, mobile communication also played a part in increasing living cost. Therefore, it is safe to say that the Covid-19 pandemic has brought economic hardships in most families across Indonesia.

In relation to FQOL, the economic impact seems to have a direct relation with the way families have to adapt. Hsiao (2018) shows that parental income plays a significant role on the FQOL. Many people are less satisfied with their financial circumstances during the pandemic ( SMERU *et al.*, 2021). For others, the situation is even worse due to sudden unemployment or the closing of a business, and hence, forcing them to temporarily rely on government subsidy and aid ( Gusman, 2020; Idhom, 2020). Based on this, we expect that *the Covid-19 economic impact, marked by less income and increased expenditure, is negatively related to FQOL* (H1).

### Parental stress and lack of parental satisfaction

In general, parental stress is inherent in the parental role on a daily basis ( Cronin *et al*., 2015). The parental role includes providing care and developing intimate relationships, both of which can be exhausting and rewarding in itself. As such, it is common for parents to report some degree of parental stress in performing the role. Specifically, parental stress is defined as a set of physiological and psychological negative reactions towards the process of adaptation and demands in carrying out parental roles ( Pontoppidan *et al*., 2018). Without any specific family hardship, to some degree the stress is assumed to have no significant implication on the family. On the contrary, in times of difficulty, parental stress is likely to increase and will negatively affect parent–child relationship ( Respler-Herman *et al*., 2012). In addition, the degree of parental stress is strongly dependent on the stage of child development and the demands faced by parents ( Louie *et al*., 2017; Pontoppidan *et al*., 2018).

In looking further at parental stress, one should also consider it from the perspective of how parents respond to their stressful situations ( Cronin *et al*., 2015). This becomes a key for parents in managing the stress they experience and finding effective coping strategies. Specifically, when parents perceive the role of parents as rewarding, for example through happiness, enjoyment, optimism, intimacy and satisfaction with the child, then they are likely to reap satisfaction from performing the parental roles ( Berry & Jones, 1995). Those with high parental satisfaction are expected to enjoy performing their role as parents, enjoy being close to children and can show positive emotions when they are with the children. On the other hand, parents who perceive parenting as burdensome and demanding are expected to have a high degree of stress to the extent that they have difficulty in recognizing their children’s basic needs ( Respler-Herman *et al*., 2012).

In line with this, parental stress scale (PSS) was developed as a way to determine the extent of parental stress in carrying out parenting role ( Berry & Jones, 1995; Pontoppidan *et al*., 2018). This is measured through the dichotomy of the parental stress subscale and the parental satisfaction subscale. PSS has been adapted in various countries such as China, Spain, Portugal, Denmark, Malaysia, and many others ( Pontoppidan *et al*., 2018). Given the fact that the Covid-19 pandemic has brought many difficulties to a large number of families in Indonesia, it is likely that the level of parental stress is increased while the parental satisfaction is decreased. Events, such as supervising child (ren) while working at home, being restricted to indoor activity, are likely to cause disturbances among parents ( Griffith, 2020). Therefore, in times of the Covid-19 pandemic, *we expect that parental stress is negatively related to FQOL* (H2).

### Family resilience

Resilience has been widely studied both at the individual as well as at the family levels ( Masten, 2019). While the former level refers to individual’s capacity to ‘bounce back’ from difficulty ( Smith *et al*., 2008), the latter refers to the capacity of a family system to survive and emerge from unfavourable circumstances, and to get stronger and more empowered ( Walsh, 2003). This definition is in line with the conception of family as an adaptive system and a context for human development.

Family resilience has extended the development of theory in the field of family stress, coping, and adaptation ( Patterson, 2002). During difficult times, family resilience will manifest itself in the extent to which a family is able to fulfil family functions, i.e., family membership, economic support, nurturance, and protection ( Li *et al*., 2019). We can claim that family resilience is a buffering factor in which families can rely on in times of hardship. However, it is important to note that there are times when families are able to swiftly bounce back from one adversity but take longer to recover from another type of adversity. This suggests that family resilience is also a process of continual growth and change across the life-span ( Walsh, 2003).

Furthermore, Walsh (2003, 2016) identify three key processes within domains of family functioning to define family resilience. The dimensions are (1) family belief system which consists of sub-components of making meaning of adversity, positive outlook, transcendence and spirituality; (2) organizational processes that consists of flexibility, connectedness, and mobilizing sources and economic resources sub-components; and (3) communication/problem-solving processes consisting of clarity, open emotional sharing, collaborative problem-solving. The three key processes enable families to work together in times of great stress in order to fulfil family functions mentioned earlier ( Prime *et al*., 2020).

Specifically, it is argued that family belief systems reflect by and large how families view happiness as well as adversity ( Walsh, 2003). The latter, indeed, shapes family members in their search for a meaning in times of crisis. During the Covid-19 pandemic, those with well-functioning belief system tend to look for positive motivation and encourage each other to get past the adversity. As Walsh (2016) argues, this type of family is likely to see adversity as a challenge which they can overcome with their available resources. At the same time, this way of thinking drives family members for a closer connection among each other. Through the times of the pandemic, well-functioning families show flexibility in adapting to new challenges. They are likely to adapt to a new situation by turning to each other for emotional and material resources and thus, strengthening their connectedness ( Prime *et al*., 2020). Furthermore, families with well-functioning belief system and positive organizational pattern are able to show positive collaboration in times of crisis. Through connectedness, they are able to share their opinions and feelings openly ( Walsh, 2003). This open communication allows family members to create effective decision-making process and hence, they are likely to operate effectively in their efforts to solve problems. On the whole, the combination of three key process of family resilience is a buffer to protect family functioning in times of crisis. Therefore, we hypothesize that *family resilience positively moderates the relation between Covid-19 economic impact and FQOL* (H3) *.* Similarly, parental stress is likely to be heightened due to unprecedented social policies implemented during the Covid-19 pandemic. However, taking into account the potential buffering effect of family resilience, we expect that *it positively moderates the relation between parental stress and FQOL* (H4) *.*

### Individual characteristics

We employ demographic information to further explore the FQOL. Typical information such as gender, age, educational level, and number of child(ren) are included as individual’s characteristics. We also take demographic information as control variables to help ensure that there are no spurious relationships when considering the relevant predictors.

## Methods

Prior to the study, we have carefully reviewed our steps of data collection to ensure that we conform to the ethical principles of psychology and social science fields. The ethical committee of the Faculty of Psychology where the authors are affiliated have approved the ethical clearance for the questionnaire and the survey (3/Psy/2021 on 1 June 2021). In addition, we have published the protocol for this study and it is publicly available ( dx.doi.org/10.17504/protocols.io.3byl4jbz2lo5/v1). The protocol, along with the measures employed in this study, can be used for replication and future studies. In this section, we start by explaining participants involved and continue to measures employed in the study. Finally, we conclude by delineating our strategy for analysis to answer the proposed hypotheses.

### Participants

This study was conducted online from August 2021 until October 2021 using Qualtrics covering a wide coverage of Indonesia. This was done by involving local enumerators from several big cities, *i.e.*, Bandung, Jakarta & its vicinities, Makassar, and Ambon, and instructed them to purposively distribute the Qualtrics link to their networks in local universities. Employing networks of local universities enable us to reach alumni and their extended networks. Although we did not aim for a nationwide generalizability, we believe that the selection of these cities represent a relevant case of Covid-19 impact on mostly urban areas (see Gusman, 2020; Idhom, 2020; Radhitya *et al*., 2020). Our selection criteria of participants were (1) a parent who lives together with their child(ren) and with or without their spouse or partner, and (2) has lived in their city of residence for at least two years. We acknowledge that there are limitations to our purposive sampling strategy, such as sampling representativeness. However, due to the Covid-19 measures, this was the best option to pursue in gathering participants from various locations. We also acknowledge that our participants would be biased towards middle income family due to the requirement of internet connection, accessible device and an intermediate skill of operating it. For this, sociodemographic information will be treated as control variables in the main analysis to ensure that there are no spurious relationships.

In a span of 3-month period, we distributed the survey link and successfully recruited 212 participants. The survey took about fifteen to twenty minutes to complete. In the end, we did not compute the time average taken by participants, because we set the online survey to allow participants to take a break during their participation thus, allowing multiple sessions or even days to finish the full questionnaire. The participants did not receive reward in any form. From 212 participants, we were only able to involve 169 participants due to either a substantial number of missing values or incomplete survey after 2 months. One from Takengon, two from Medan, 66 from Bandung and its vicinities ( *e.g.*, Cimahi, Garut, Sumedang, Tasikmalaya), 41 from Jakarta and its vicinities ( *i.e.*, Bekasi, Bogor, Serang, and Tangerang), 18 from Semarang and its vicinities (Cilacap, Yogyakarta, and Kebumen), four from Palangkaraya and its vicinities ( *i.e.*, Tarakan and Pontianak), seven from Surabaya and its vicinities ( *i.e.*, Nganjuk, Rembang, and Sampit), three from Denpasar and its vicinity ( *i.e.*, Labuan Bajo), nine from Makassar and its vicinities ( *i.e.*, Sengkang and Manado), 17 from Ambon, and finally one from Timika Papua. Table 1 provides the overview of our participants.

**Table 1.** Descriptive statistics of all variables.

	Range	Min	Max	Mean	SD
**Predictors**					
Family quality of life	1–5	1.83	5	3.95	0.58
Covid-19 economic impact	1–3	1.50	3	2.43	0.43
Parental stress	1–5	1	3.29	1.82	0.50
Family resilience:	1–5	1.77	5	3.95	0.50
- Belief system	1–5	2.55	5	3.93	0.53
- Organizational pattern	1–5	1	5	3.97	0.61
- Problem solving	1–5	1.30	5	3.93	0.62
**Individual characteristics**					
Age	18–65	18	64	47.06	11.19
Gender (female coded 0)	0/1	0	1	.31	0.46
Educational level	1-6	4	6	5.27	0.59
Number of child(ren)	-	1	5	2.27	0.95

Furthermore, we looked more closely at the demographic information. Table 2 provides a detailed account of the information. First, in line with the mean value of the number of children ( *M*=2.27), most participants (44%) from our sample are shown to have 2 children. Second, we show that, on average, most participants (48.5%) experienced a decreased income during the Covid-19 pandemic. Although we notice that there is also a considerable number of participants (44.4%) who reported no change in their income. Meanwhile, a large number of participants (56.2%) reported an increased expense during the pandemic. Lastly, we also gathered a work status of participants and classified them into three broad categories, namely employee, entrepreneur and housewife/husband. It is shown that most of our participants (49.7%) are employees and the remaining are distributed almost equally across the other two categories. As a follow-up question, we asked participants whether there was a change due to the Covid-19 pandemic. The answer categories are 1 “no change”; 2 “instructed to stay home with a pay cut” for employees, “operational/economic capacity reduced to 50%” for entrepreneur and housewife/husband; 3 “instructed to stay home without pay” for employees, “operational/economic capacity reduced to more than 50%”; and 4 “laid off” for employees, “forced to close down” for entrepreneur and “economic capacity becomes impoverished” for housewife/husband. Here, a big majority of our employee and housewife/husband samples reported no change in their work and family. On the other hand, in the entrepreneur sample group, most participants (65.1%) reported that they had to reduce their operation by 50% or even reduced the operation to more than. While a small number of employee (1.2%) and entrepreneur (11.6%) reported to have been laid off or forced to close down their business.

**Table 2.** Frequency of demographic characteristics.

Variables	Frequency (%)			
	1	2	3	4 (and more)
Number of child(ren)	35 (20.7)	75 (44.4)	39 (23.1)	20 (11.9)
Change of income *	10 (5.9)	77 (45.6)	82 (48.5)	
Change of expense *	23 (13.6)	51 (30.2)	95 (56.2)	
Work status **	84 (49.7)	43 (25.4)	42 (24.9)	
(1) Employee	74 (88.1)	9 (10.7)		1 (1.2)
(2) Entrepreneur	10 (23.3)	13 (30.2)	15 (34.9)	5 (11.6)
(3) Housewife/husband	26 (61.9)	10 (23.8)	6 (14.3)	

* Higher score indicating higher impact.

** 1=employee; 2=entrepreneur; 3=housewife/husband.

### Consent to participate

Prior to filling in the questionnaire, the participants were given brief information of the study. Next to that, they were required to give an informed consent that they have been given sufficient time to carefully read the study information and there was no undue influence on their decision to participate. If agreed, participants were allowed to move on to the questionnaire. Otherwise, they would be directed to the end of the survey. No ‘consent for publication’ was necessary as the survey did not require personal information, such as name, initial, and other traceable information. We also disabled the recording of the internet protocol (IP) address on the Qualtrics survey to ensure that no unnecessary personal information was recorded. In terms of data management, only the research team had access to the dataset excluding the local enumerators.

### Measures

We employed measures that have been tested in previous studies. To serve the purpose of this study, we translated the measures into Indonesian language. Specifically for parental stress and family resilience measures, we checked our translation with the Indonesian studies that have involved the measures previously. Our translation is similar to the previous studies. As for the FQOL measure, each of us translated the measure and compared the results. Subsequently, we had to reach a consensus on the selected translation for each statement.

Next, to ensure their applicability in our research context, we conducted confirmatory factor analysis (CFA) using lavaan package in R version 4.2.1 (RRID:SCR_001905; https://lavaan.ugent.be/). In addition, we calculated *Alpha Cronbach* to assess the internal consistency of all the measures and average variance extracted (AVE) and composite reliability (CR) to measure the amount of variance that is accounted for by the latent constructs ( Fornell & Larcker, 1981). The conventional benchmark for reliability level is larger than 0.70 or 0.80 ( Brunner & Süß, 2005). As for AVE, the value should be larger than any correlation found between any pair of latent constructs or minimum at 0.50 ( Fornell & Larcker, 1981). However, when AVE is found to be lower than 0.50 but CR is higher than 0.60, Fornell and Larcker (1981) argue that due to a more conservative estimate of convergent validity a construct with AVE lower than 0.50 can still be considered valid. Table 3 provides a bivariate correlation of the variables along with the value of AVE.

**Table 3.** Bivariate correlations among predictors.

Variables	FQOL	Covid-19 impact	Parental stress	Family resilience	Belief system	Organizational pattern	Problem solving	Age	Educational level	Number of children
FQOL	-	−0.08	− **0.36**	**0.66**	**0.61**	**0.44**	**0.65**	0.12	−0.04	0.01
Covid-19 impact		-	−0.11	0.04	0.04	0.00	0.04	0.05	−0.10	0.01
Parental stress			-	− **0.26**	− **0.32**	−0.10	− **0**.22	−0.02	0.03	0.13
Family resilience				-	**0.83**	**0.87**	**0.87**	0.04	−0.03	−0.06
- Belief system					-	**0.60**	**0.60**	−0.15	0.01	−0.11
- Organizational pattern						-	**0.63**	−0.00	0.00	−0.05
- Problem solving							-	0.11	−0.08	−0.06
Age								-	−0.08	**0.25**
Educational level									-	−0.09
Number of children										-
**AVE**	0.36		0.38	0.31	0.31	0.27	0.36			
**CR**	0.90		0.89	0.92	0.75	0.77	0.85			

* Bold indicates significance at 0.05.

### Family quality of life

As mentioned earlier, we employed a measure of FQOL by Beach Center on Disability ( Hoffman *et al*., 2006). The scale assesses family member’s perception towards the different aspects of family life. The original scale consists of five domains, namely family interaction, parenting, emotional well-being, physical/material well-being, and disability-related support. There are 25 items in the original scale, but with the exclusion of disability-related dimension we employed 19 items spread across four dimensions. Family interaction is assessed through items such as, “My family enjoys spending time together”. Parenting is measured by items such as, “Family members help the children learn to be independent”. Emotional well-being is assessed through items such as, “My family has the support we need to relieve stress”. Finally, material well-being poses items such as, “My family has a way to take care of our expenses”. All items are rated on a five-point Likert scale, with 1 being very dissatisfied and 5 being very satisfied.

Based on the CFA results, we observe that the measurement model is indeed composed of four dimensions. However, the first model did not show a good fit with the data. We noticed that one item from each dimension of material well-being, family interaction, and parenting shared a substantial variance with other dimensions. After the removal the CFA shows a better fit model, Chi-squared = 191.94, p < 0.001, confirmatory fit index (CFI) = 0.94, root mean square error of approximation (RMSEA) = 0.08, and the standardized root mean squared error (SRMR) = 0.04 (see Appendix 1 in the extended data for a final list of items). According to Hooper *et al*. (2008), such fit measures are considered as a good fit model. The factor loadings of the remaining items range from 0.47 to 0.72, indicating a good level of accounted variance ( Peterson, 2000). In addition, the dimensions of the scale have a high level of reliability, with α = 0.89 for family interaction, α = 0.87 for parenting, α = 0.81 for emotional well-being and finally α = 0.74 for material well-being.

### Covid-19 economic impact

According to a report by SMERU *et al*. (2021), Covid-19 has brought economic challenges to many families across Indonesia. They based their conclusion on two main economic indicators, namely reduced income and increased expense. Therefore, to assess the economic impact brought by the Covid-19 pandemic, we asked two items regarding respondents’ change of income and expense. Respondents were asked to rate the items on a three-point Likert scale. The option categories range from 1 being income/expense is reduced to 3 income/expense is increased. Later in the analysis, we reversed the answer on a change of income item and calculated the total score for both items. Due to a two-item scale, we calculated its reliability by running a *Pearson* correlation and the result shows a significant correlation ( *r =* 0.17, *p* = 0.02).

### Parental stress

Parental stress scale (PSS) was employed to determine the extent of parental stress in carrying out parenting role during the Covid-19 pandemic ( Berry & Jones, 1995; Pontoppidan *et al*., 2018). The scale is composed of two subscales, that is the level of parental stress and the parental satisfaction. Parental stress subscale asks respondents to rate their perception towards their parental role through items, such as “Having children has been a financial burden”. While parental satisfaction asks respondents to rate their satisfaction towards their parental role through items, such “I feel close to my child (ren)”. All items combined make up a total of 18 items and are rated on a five-point Likert scale, with 1 being strongly disagree and 5 being strongly agree. To compute the scale, we reversed the answer on all items in parental satisfaction subscale and then calculated the total score based on all items.

Through CFA, the first model shows a poor fit model. We observed that four items of parental stress subscale and one item of parental satisfaction subscale had a substantial shared variance with another subscale. After the removal of those items, the CFA shows a good fit model, Chi-squared = 116.89, p < 0.001, CFI = 0.95, RMSEA = 0.07, and the SRMR = 0.05. The factor loadings range from 0.51 to 0.71, indicating an acceptable level. The two subscales show a high level of reliability, α = 0.88 for parental stress and α = 0.90 for lack of parental satisfaction.

### Family resilience

We assessed family resilience by employing family resilience framework by Walsh (2003). The framework proposes a multidimensional scale of family resilience, consisting of belief systems, organizational pattern and communication/problem solving ( Walsh, 2016). Belief systems asks respondents to rate themselves on statements, such as “We try to understand the stress situation and focus on our choice”. Organizational pattern consists of statements on family’s flexibility and the way they organize themselves in times of crisis, such as “We are flexible in adapting to a new challenge”. Finally, communication/problem solving assesses respondents’ way of communication in a family through statements, such as “We can express our opinion and be honest to one another”. Similar to previous scales, all items are rated on a five-point Likert scale, with 1 being strongly disagree and 5 being strongly agree. In total, the original scale consists of 32 items.

The first model in our CFA shows a poor fit model. Four items from belief systems dimension, and one item each from organizational pattern and communication/problem solving dimensions should be reconsidered due to cross loadings and low factor loading. After the removal of those items, the CFA demonstrates a good fit model, Chi-squared = 443.34, p < 0.001, CFI = 0.94, RMSEA = 0.06, and the SRMR = 0.05. The factor loadings of the remaining items range from 0.46 to 0.73, showing an acceptable level. Finally, all the dimensions of the scale show a high level of reliability, α = 0.91 for belief system, α = 0.93 for problem solving and α = 0.82 for organizational pattern.

### Individual characteristics

We employed straightforward demographic questions concerning age, gender, educational level, and the number of child(ren). These items help us in the main analysis to ensure that there are no spurious relationships of interest when individual characteristics are factored in the statistical models.

### Strategy for analyses

Prior to running the main analyses, we conducted preliminary tests to ensure that our data fit the statistical assumptions, *i.e.*, linearity, normal distribution, and multicollinearity. The ANOVA tests show that most of our predictors have a linear relationship with the FQOL. Moreover, the values of skewness and kurtosis of all our variables are in the range of −0.40 to 0.58 to −0.71 to 1.46, respectively, which suggest an acceptable range of normal distribution ( Kim, 2013). Finally, the multicollinearity tests show that the values of variance inflation factor (VIF) and tolerance statistics are within acceptable range, that is from 1.01 to 1.08 and 0.93 to 0.99, respectively ( Field, 2009; O’Brien, 2007). Based on all this, we can safely continue to our moderation analysis. In doing so, we mean-centred the predictors to enable easier interpretation on the results, *i.e.*, high level of family resilience refers to above the mean and low level refers to below the mean.

## Results

We ran a moderation analysis in a step-wise fashion. Table 4 provides the full results of the analysis. In Model 1, we included only the main predictors of Covid-19 economic impact and parental stress. Here, although the relation of Covid-19 economic impact with FQOL is negative, it is not significant enough to affect FQOL ( *b =* −0.12, *p =* 0 *.*22). Furthermore, Model 1 shows that there is indeed a significant negative relation between parental stress and FQOL ( *b* = −0.46, *p <* 0.00). Based on Model 1, we can conclude that the results disconfirm a hypothesis on the negative relation between Covid-19 economic impact and FQOL (H1) but fully supports a hypothesis on the relation between parental stress and FQOL (H2).

**Table 4.** Conditional effect of family resilience on the relation between predictors and FQOL (N = 158), with standard error in parentheses.

Predictors	Model 1	Model 2	Model 3	Model 4
Intercept	**3.94**	**3.95**	**3.96**	**3.75**
Covid-19 economic impact	−0.12	−0.14	−0.13	−0.13
Parental stress	− **0.46**	− **0.27**	− **0.27**	− **0.29**
Family resilience		**0.69**	**0.78**	**0.77**
*Interaction effect*				
Covid-19 economic impact *Family resilience			**0.32**	**0.31**
Parental stress *Family resilience			0.22	0.20
*Individual characteristics*				
Age				0.01
Gender (female = 0 as reference)				0.08
Educational level				−0.02
Number of child				0.02
*Adjusted R ^2^*	**0.14**	**0.49**	**0.52**	**0.52**

* Bold indicates significance at 0.05.

Subsequently, Model 2 adds family resilience into the model. Here, it shows that there is a substantial positive relation between family resilience and FQOL ( *b =* 0.69, *p <* 0.00). This result indicates a plausible conditional effect of family resilience on the relation between the predictors and FQOL. However, the interaction effect should be tested further. Hence, we continue to Model 3 and Model 4.

In Model 3, with the inclusion of interactions between Covid-19 economic impact & parental stress and family resilience, we demonstrate that the significance of main effects remain similar ( *b =* −0.27, *p <* 0.00 for parental stress; *b =* 0.78, *p <* 0.00 for family resilience). Further, we notice that there is a positive interaction between Covid-19 economic impact and family resilience on FQOL ( *b =* 0.32, *p =* 0.02). On the contrary, there is no significant interaction between parental stress and family resilience on FQOL ( *b =* 0.22, *p =* 0.08). Therefore, the results partially confirm the moderation hypotheses. We confirm that family resilience positively moderates the relation between Covid-19 economic impact and FQOL (H3), however, we reject the notion that family resilience positively moderates the relation between parental stress and FQOL (H4).

**Figure 1.**
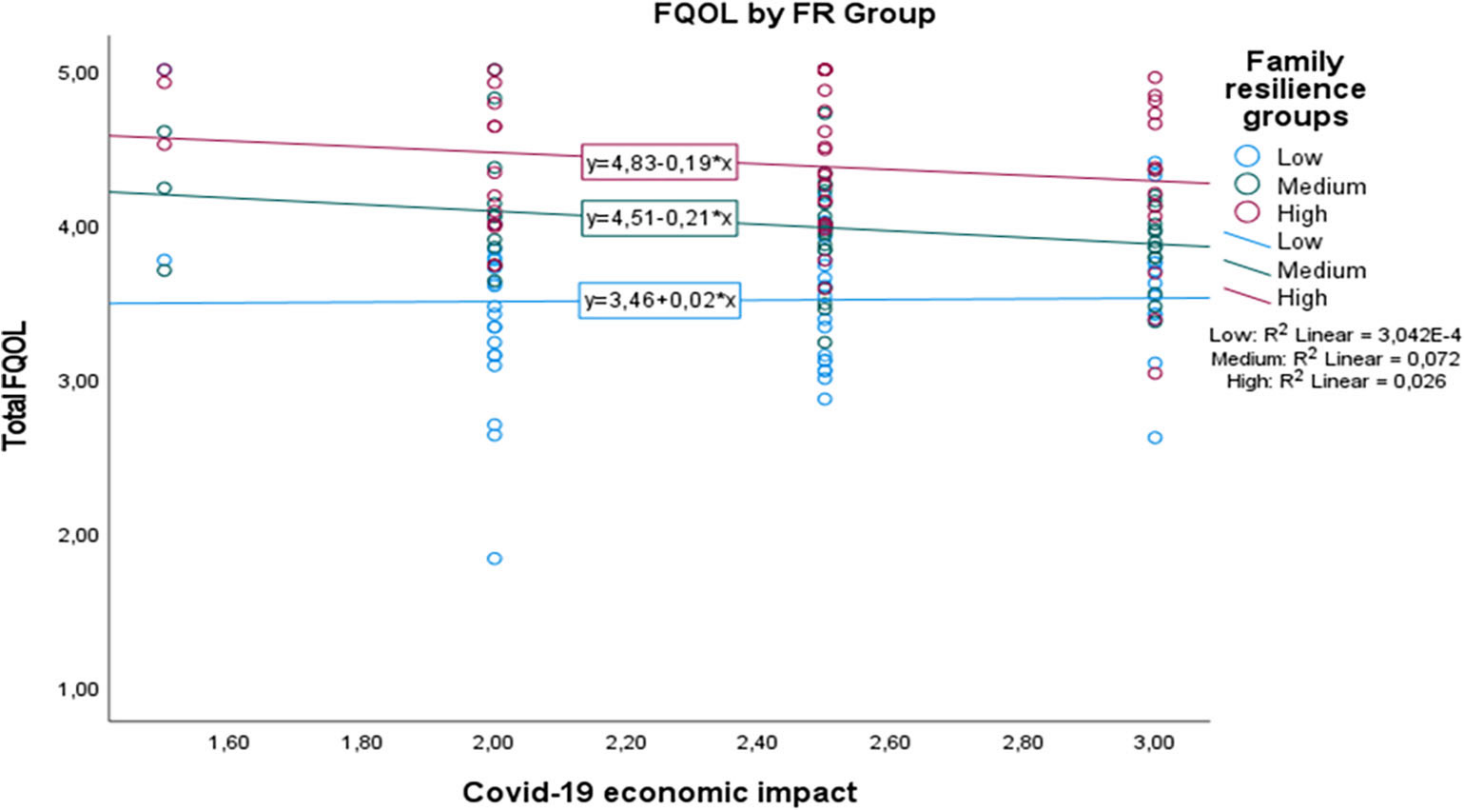
The conditional effect of family resilience on the relation between Covid-19 economic impact and FQOL.

**Figure 2.**
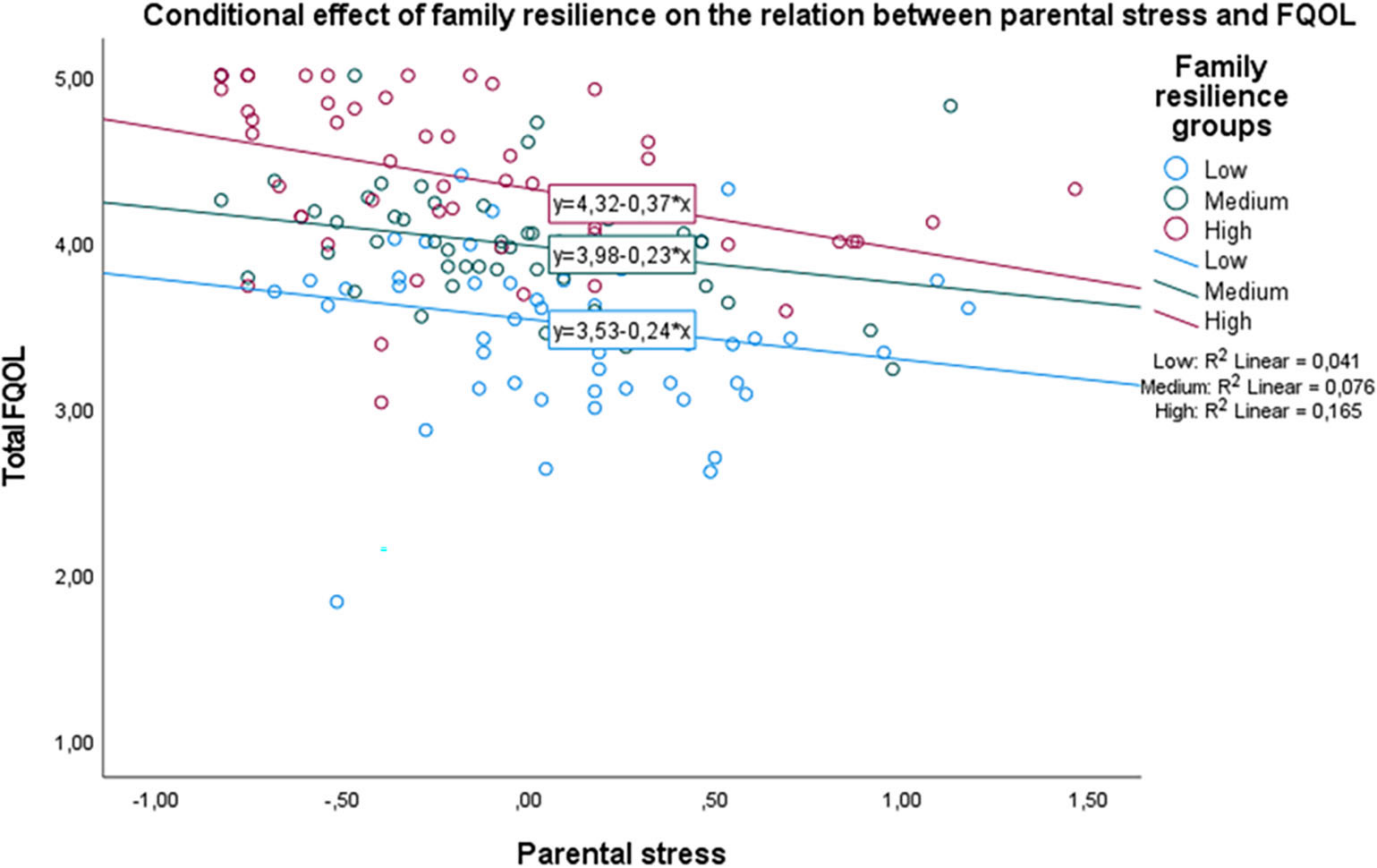
The conditional effect of family resilience on the relation between parental stress and FQOL.

Figure 1 shows that although the medium and high family resilience groups seem to show a decrease in FQOL, here, we clearly see that those with medium to high family resilience level are better positioned in their quality of life in relation to their perception towards Covid-19 economic impact. Interestingly, those with low family resilience level are likely to show an increase in their FQOL when they perceive higher Covid-19 economic impact. On the other hand, Figure 2 shows that the main effect of parental stress is large enough to hinder the positive effect of family resilience on FQOL. Regardless of family resilience levels, those who perceive high parental stress are likely to show a substantial decrease in their FQOL.

Finally, in Model 4, we made sure that our previous relations do not substantially change by including demographic characteristics. Here, we show that demographic characteristics appear to have no significant relation with FQOL and at the same time, the previously found significant relations do not substantially change. Therefore, we can safely assume that the significant relations between parental stress & family resilience, on one hand, and FQOL, on the other, are not affected by individual characteristics.

## Discussion and conclusion

The Covid-19 pandemic unquestionably has become an unparalleled challenge for families across the world, especially in Indonesia. Taken into account the most notably effect of the pandemic, namely economical and psychological impacts, we aim to answer to what extent the Covid-19 economic impact & parental stress during the pandemic are related to FQOL while moderated by family resilience in several cities of Indonesia. The results show mixed findings, with most of the proposed hypotheses are supported.

First, although many reports show that the economic impact brought by the Covid-19 pandemic hit hard many families across the income level ( SMERU *et al*., 2021), we do not find that this impact is negatively related to FQOL among our respondents. This conflicts with other previous studies that show when family’s material well-being is affected, such as due to unemployment change, their family interaction and parenting are more likely to be reduced ( Tsai & Chen, 2017). Hence, the low FQOL is assumed. We offer three reasons regarding this finding. One, according to many reports, local governments were claimed to be cooperative efficiently with the central government of Indonesia in delivering aid to those who have lost employment and substantially reduced income due to the pandemic ( Hanaf *et al*., 2020). In addition, several social assistance programs, such as the Family Hope Program ( *Program Keluarga Harapan*; PKH) and food staples program through grocery card ( *Kartu Sembako*) were quickly modified not only aimed for the lowest-income families but also for new beneficiaries who were affected by the pandemic ( SMERU *et al*., 2021). In several areas, there were even cash transfer programs directly to the households. Therefore, although the economic impact was heavily felt families were still able to cope with economic assistances from the government. Two, closely related to the delivery time of the assistances, this study was conducted in the middle of 2021 where many government aids have started to be delivered ( Saptoyo, 2021). Thus, the Covid-19 economic impact may have worn down and families have adapted to their new financial circumstances. Three, we notice that most of our participants did not report a substantial decrease in income due to the pandemic. This is also in line with a large number of employees and housewife/husband (and a considerable number of entrepreneur) who reported no change in their work or business status. This number may explain why we find no evidence on the relation between economic impact and FQOL in our samples.

On the contrary, the assessed parental stress level during the pandemic is shown to be significantly related to FQOL. This is in line with the claim that impact of parental stress can spill-over to their relationship with the children and their children’s well-being ( Berry & Jones, 1995). In this situation, the Covid-19 pandemic is considered to be a major stressor for the parents and hence, affecting their behaviours towards their children. Studies have shown that parents have a higher chance to experience burnout and perform ‘bad’ parenting, such as children maltreatment ( Chung *et al*., 2020; Griffith, 2020; Lawson *et al.*, 2020). As a consequence, the relationship between parents and child (ren) are impaired and most importantly, negatively affect their FQOL ( Summers *et al*., 2005).

Next, we have shown that family resilience is positively, directly, related to FQOL. As Prime *et al*. (2020) argue, family resilience should foster positive outcomes in times of Covid-19 among family members. Although the quality of relationships are most likely to be affected during the pandemic, those with high level of family resilience should be able to improve and maintain quality family relationships. Apart from that, Walsh (2016) suggests that in times of crisis such families will develop new ways of viewing challenges and thus, encouraging positive outlook out of the crisis.

In addition, we should also take into account by which family systems work in Indonesia. Reciprocation between parents and children are still regarded highly in the Asian context, including Indonesia ( Tsai & Chen, 2017). In times of crisis, this tendency can be a valuable resource in which parents with no hesitation financially assist their children or otherwise. Such family-reliance behaviours can also extend to co-residing with either or both parents. This norm, therefore, may play a role in maintaining the way family members communicate and support each other. In turn, their family quality of life is well protected from being drastically affected by the pandemic.

Furthermore, we have also confirmed the moderating effect of family resilience on the relation between the family stressor and FQOL. Specifically, on average, we find that our respondents are economically impacted by the Covid-19 pandemic (see Table 1). Although the impact is not related to FQOL, with family resilience we can claim that their FQOL is relatively maintained and even increased, even for families with a low level of family resilience. This finding corroborates previous studies that show family resilience help families in developing new ways of dealing with challenges relying on resources at their disposal ( Li *et al*., 2019). Through well-functioning ways of seeing a crisis or challenge, flexibility among family members, and open communication, they can rely on each other and create a better decision-making in attempting to ameliorate their momentarily-deprived economy ( Walsh, 2003). As a consequence, family resilience may foster positive psychological outcomes in times of crisis. However, this claim should be further studied since our study does not test any causality.

In spite of the direct relation between family resilience and FQOL, the buffering effect of family resilience is shown to be less effective in moderating the negative relation between parental stress and FQOL. This finding opens alternative explanations to the literature of family resilience as well as parental stress. First, parental stress during crisis times is highly likely to be heightened and thus, largely affect the way families navigate themselves in solving the crisis. Therefore, even when protective factors are present within the family, such as positive family relationships, parental stress will most likely still reduce many aspects of the quality of family life, such as parenting and family well-being ( Tsai & Chen, 2017; Zuna *et al*., 2009b). Second, it shows a persisting effect of parental stress during crisis times. Unlike economic impact of Covid-19, parental stress seems to persist even when the pandemic has turned to one year. Keeping the Covid-19 pandemic situation in mind, this is logical because psychological help, extended family members’ help, and even neighbours’ help is still very limited due to health protocols during the pandemic. Therefore, parental stress is likely to persist in affecting FQOL. Finally, the finding opens a possible discussion that family resilience should be considered as a process and may work differently in different stages of crisis. Similar to the notion of resilience in general, family resilience should be viewed in its temporal context during the times of crisis ( Masten, 2019).

It is also worth discussing why demographic characteristics are not related to the FQOL. First, by the time the survey was underway, most family members, irrespective of their gender, education and a number of children, were experiencing a similar change in their quality of life. After all, the Covid-19 pandemic has been shown to affect most, if not all, families in Indonesia ( SMERU *et al.*, 2021). Second, quality of life of an individual has been shown to be mainly related to their relations with others ( White, 2015). Therefore, rather than their individual characteristics, the extent to which individuals develop their social relations positively may, indeed, play a more significant role in determining their FQOL. Thus, its relation between family resilience is shown to be very strong in this study. In light of this finding, we encourage other scholars to further investigate the notion of FQOL in other adverse circumstances, while taking into account the localities of the research area. Contextual factors, such as religiosity and ethnic identification, may play a role in developing FQOL among families in Indonesia ( Cameron, 2004; see Hadiz, 2017 for the importance of religious identity).

We acknowledge several limitations in this study. One, we realize that our small sample may not reflect the whole picture of the family situation across Indonesia. and therefore, we cannot infer anything about local cultural differences across the regions. These differences, of course, might have played a role in determining the level of FQOL. In addition, conducting online survey in times of the pandemic apparently remains a challenge to many Indonesians, ranging from ‘too exhausted viewing screen’, less enthusiastic, low internet coverage, to a lack of skills in operating the application ( Wisanti *et al*., 2021). The anticipation of ‘online exhaustion’ also encouraged us to reduce a number of questions, such as years of marriage and co-residing with parent(s), so that we would still have a high completion rate. Therefore, not only were we unable to acquire a desired sample size, we were also unable to cover an extensive list of demographic questions. Two, the study was a one-shot study. We do not have data on the variables of interests prior to the pandemic. Therefore, we cannot compare the disparity between the two conditions. Most importantly, we cannot draw any causal conclusion regarding the relations under study. We know that there is a different dynamic in the relationships between parental stress and family resilience and FQOL during the pandemic, but we cannot be sure to what extent the pandemic has brought changes to these relationships. Finally, in adapting the English scales into Indonesian questionnaire we did not perform a back-translate to the original language and have experts rate the differences between the Indonesian and English versions. Although in our case the Indonesian translation was similar to previous studies in the Indonesian context, we still encourage future studies to undergo a back-translate procedure to verify that the original meaning is maintained after the translation.

In conclusion, the findings of the study shed a light into family circumstances in Indonesia during the Covid-19 pandemic, which has not received adequate academic attention. We show that after a one-year experience of Covid-19, on average, respondents do not feel that the economic impact is negatively related to their FQOL. However, unlike the economic impact, parental stress is negatively related to the FQOL. This might be due to the many changes they had to undergo during the pandemic which has exacerbated their parental stress level. On a positive note, we find that family resilience can help families buffer the economic impact of the Covid-19 pandemic on their FQOL, although not so much with parental stress. Based on this, government agencies and other related parties should put more attention to offering psychological help to families through available mediums, *e.g.*, social media and hotline numbers, that are Covid-19 proof. Finally, the current findings suggest that unexpected challenge or a sudden change is inevitably part of every family life. One way to overcome this is by adapting family resilience as a system, in which the adaptation is largely dependent on the way family members utilize their relationship and exchange of social support ( Masten, 2019). By this, family members can rely on each other and at the same time overcoming their individual limitation. Based on this, future studies are encouraged to pursue the relevance of family resilience in overcoming other challenging events, such the loss of a family member, juvenile delinquency and families of child(ren) with a disability.

## Author’s note

This research adheres to research ethics provided the Committee on Publication Ethics (COPE).

## Data Availability

Figshare: Underlying data for “The conditional effect of family resilience on family quality of life during the Covid-19 pandemic”. DOI: https://doi.org/10.6084/m9.figshare.20746411 ( Setiawan *et al.*, 2022). Figshare: Extended data for “The conditional effect of family resilience on family quality of life during the Covid-19 pandemic”. DOI: https://doi.org/10.6084/m9.figshare.21342126 ( Setiawan *et al.*, 2022). Data are available under the terms of the Creative Commons Attribution 4.0 International license (CC-BY 4.0).
