# Supplementary material for: The conditional effect of family resilience on family quality of life during the Covid-19 pandemic
Source: F1000Res. 2023 Jan 12;11:1279. Originally published 2022 Nov 9. [Version 3] doi: 10.12688/f1000research.125852.3 (PMC9830109; doi:10.12688/f1000research.125852.3)
Supplement: Peer review history

# Peer review: Reviewer response for version 3

**DOI:** 10.5256/f1000research.143011.r160060

**Published:** 2023-01-13

**Authors:** Christiany Suwartono

**Affiliations:** 1 Faculty of Psychology, Atma Jaya Catholic University of Indonesia, Jakarta, Indonesia; 2 Pusat Unggulan Kebijakan Kesehatan dan Inovasi Sosial (PUI-PT PPH), Universitas Katolik Indonesia Atma Jaya, Jakarta, Indonesia

I have reviewed the revised manuscript, and I believe that the authors have satisfactorily addressed all of the comments and concerns that I raised in my review.

Is the work clearly and accurately presented and does it cite the current literature?

Yes

If applicable, is the statistical analysis and its interpretation appropriate?

Partly

Are all the source data underlying the results available to ensure full reproducibility?

Partly

Is the study design appropriate and is the work technically sound?

Yes

Are the conclusions drawn adequately supported by the results?

Yes

Are sufficient details of methods and analysis provided to allow replication by others?

Yes

Reviewer Expertise:

social psychology, psychometrics, forgiveness, and positive psychology.

I confirm that I have read this submission and believe that I have an appropriate level of expertise to confirm that it is of an acceptable scientific standard.

# Peer review: Reviewer response for version 2

**DOI:** 10.5256/f1000research.141977.r157992

**Published:** 2023-01-09

**Authors:** Christiany Suwartono

**Affiliations:** 1 Faculty of Psychology, Atma Jaya Catholic University of Indonesia, Jakarta, Indonesia; 2 Pusat Unggulan Kebijakan Kesehatan dan Inovasi Sosial (PUI-PT PPH), Universitas Katolik Indonesia Atma Jaya, Jakarta, Indonesia

I have reviewed the revised manuscript, and I believe that the authors have satisfactorily addressed all of the comments and concerns that I raised in my review.

 The revised manuscript is now ready for indexing in my opinion. I believe that the changes made by the authors have significantly improved the quality and clarity of the manuscript, and I am confident that it will be of interest to the readers of your journal.

Is the work clearly and accurately presented and does it cite the current literature?

Yes

If applicable, is the statistical analysis and its interpretation appropriate?

Partly

Are all the source data underlying the results available to ensure full reproducibility?

Partly

Is the study design appropriate and is the work technically sound?

Yes

Are the conclusions drawn adequately supported by the results?

Yes

Are sufficient details of methods and analysis provided to allow replication by others?

Yes

Reviewer Expertise:

social psychology, psychometrics, forgiveness, and positive psychology.

I confirm that I have read this submission and believe that I have an appropriate level of expertise to confirm that it is of an acceptable scientific standard.

# Peer review: Reviewer response for version 2

**DOI:** 10.5256/f1000research.141977.r157993

**Published:** 2022-12-13

**Authors:** Tita Alissa Bach

**Affiliations:** 1 Group Technology and Research, Life Sciences program, Healthcare Programme, Det Norske Veritas (DNV), Oslo, Norway

Thank you for the revision. I have just minor comments that you may choose to address. Otherwise, a very well written paper and I hope that others can learn from your study not only during a pandemic. 

 Abstract:

- Spell out CFA?
- What does this mean: "family resilience only positively moderates.."?

Method:

- Translating the questionnaire into Indonesian without back-translate it to English and rate differences between the two (original in English vs. back-translated English), which is a standard procedure in a survey study, is okay for your study, but I would suggest to put this as a limitation.

Conclusion:

- I think your study is important and not only during the pandemic. If possible, it would be very helpful for other researchers to provide future directions on how your study can be made relevant for situations other than just during a pandemic. 

Is the work clearly and accurately presented and does it cite the current literature?

Yes

If applicable, is the statistical analysis and its interpretation appropriate?

I cannot comment. A qualified statistician is required.

Are all the source data underlying the results available to ensure full reproducibility?

Partly

Is the study design appropriate and is the work technically sound?

Yes

Are the conclusions drawn adequately supported by the results?

Yes

Are sufficient details of methods and analysis provided to allow replication by others?

Partly

Reviewer Expertise:

healthcare, psychology, human factors, sociology, behavioural and social sciences

I confirm that I have read this submission and believe that I have an appropriate level of expertise to confirm that it is of an acceptable scientific standard.

# Peer review: Reviewer response for version 1

**DOI:** 10.5256/f1000research.138200.r155877

**Published:** 2022-12-01

**Authors:** Christiany Suwartono

**Affiliations:** 1 Faculty of Psychology, Atma Jaya Catholic University of Indonesia, Jakarta, Indonesia; 2 Pusat Unggulan Kebijakan Kesehatan dan Inovasi Sosial (PUI-PT PPH), Universitas Katolik Indonesia Atma Jaya, Jakarta, Indonesia

The research was conducted when there was a sharp increase of Covid-19 positive cases in July - August 2021, and it declined during September 2021. The authors conducted an interesting study and the article makes an early contribution to the knowledge of family dynamics and economic conditions during Covid-19 in Indonesia - although the coverage of the geographic area in this research needs to be noted. 

 The abstract needs to be improved especially in the method section and results. 

 Keywords can also be improved - as a suggestion: covid-19 economic impact.

**Methods:**

- It was a good initiative and practice to publish the study protocol before the authors conduct the research. It allows other researchers to replicate this research.

**Demographic data:**

- The results found that Covid-19 economic impact was not significant; the authors should inform the reader more about demographic data from this sample: like the number of children in frequency (or %). A family consisting of a small number of children will have different economic burdens. Also with other categories variables: gender, and educational background. Did the authors ask about the participant's employment status?
- How about the distribution of responses regarding respondents’ change of income and expense? How many of the participants responded that their income/expense is reduced or income/expense is increased? (The authors wrote three options about this one).
- Maybe this descriptive analysis can contribute to the result explanation of this research. The authors could add one more table to describe.
- The covid-19 economic, family resilience, parental stress, and family quality of life findings were interesting. Based on the demographic data of the majority of the participants; did the authors consider that the children's age range, cultural role, and geography might influence the variables of interest?

Is the work clearly and accurately presented and does it cite the current literature?

Yes

If applicable, is the statistical analysis and its interpretation appropriate?

Partly

Are all the source data underlying the results available to ensure full reproducibility?

Partly

Is the study design appropriate and is the work technically sound?

Yes

Are the conclusions drawn adequately supported by the results?

Yes

Are sufficient details of methods and analysis provided to allow replication by others?

Yes

Reviewer Expertise:

social psychology, psychometrics, forgiveness, and positive psychology.

I confirm that I have read this submission and believe that I have an appropriate level of expertise to confirm that it is of an acceptable scientific standard, however I have significant reservations, as outlined above.

# Response

**Published:** 2022-12-07

**Authors:** Tery Setiawan

**Affiliations:** Radboud University Nijmegen, The Netherlands

We truly thank the reviewer for very insightful remarks and suggestions for the manuscript. We believe that your feedback has helped the manuscript greatly improve. The following are responses to your remarks/suggestions.

 Response to Reviewer 2 Comments:

 The research was conducted when there was a sharp increase of Covid-19 positive cases in July - August 2021, and it declined during September 2021. The authors conducted an interesting study and the article makes an early contribution to the knowledge of family dynamics and economic conditions during Covid-19 in Indonesia - although the coverage of the geographic area in this research needs to be noted.

 The abstract needs to be improved especially in the method section and results.

**Response**: Thank you for your suggestion on the abstract. We agree and therefore, have revised it accordingly.

 Keywords can also be improved - as a suggestion: covid-19 economic impact.

**Response**: Thank you for your suggestion on the keyword. We have added your suggested keyword to the list of keywords.

 Methods:

 It was a good initiative and practice to publish the study protocol before the authors conduct the research. It allows other researchers to replicate this research.

**Response**: Thank you for your encouragement. We also believe that providing protocol and materials are necessary for replication as well as further future investigations.

 Demographic data:

 The results found that Covid-19 economic impact was not significant; the authors should inform the reader more about demographic data from this sample: like the number of children in frequency (or %). A family consisting of a small number of children will have different economic burdens. Also with other categories variables: gender, and educational background. Did the authors ask about the participant's employment status?

**Response**: Thank you for your input on demographic information. We agree with this suggestion and we have provided such information in the text. We have also provided a new table as you suggested.

 In response to your question on the employment status, yes, we asked participants such information. This information is now available in the new table (Table 2)  

 How about the distribution of responses regarding respondents’ change of income and expense? How many of the participants responded that their income/expense is reduced or income/expense is increased? (The authors wrote three options about this one).

**Response**: Thank you for your feedback on providing more information of respondents’ change of income and expense. This information is now available in the new table (Table 2).

 Maybe this descriptive analysis can contribute to the result explanation of this research. The authors could add one more table to describe.

**Response**: Thank you for your suggestion on adding more information of the descriptive analysis. We have now provided a new table (Table 2) containing frequency of several demographic information to be used later in discussing the findings. Next to this, we have provided an additional explanation as to why we find no evidence on the relation between economic impact of the Covid-19 pandemic and FQOL.

 The revision is incorporated in the following part (of discussion): “Three, we notice that most of our participants did not report a substantial decrease in income due to the pandemic. This is also in line with a large number of employees and housewife/husband (and a considerable number of entrepreneur) who reported no change…”

 The covid-19 economic, family resilience, parental stress, and family quality of life findings were interesting. Based on the demographic data of the majority of the participants; did the authors consider that the children's age range, cultural role, and geography might influence the variables of interest?

**Response**: Thank you for your remark on the cultural differences. We acknowledge that local cultural differences might have played a role in determining our findings. Even though we do not have sufficient information to infer such arguments, we have provided this as an alternative explanation which, of coure, should be further investigated.

 The revision is incorporated in the following part: “In addition, we should also take into account by which family systems work in Indonesia. Reciprocation between parents and children are still regarded highly in the Asian context, including Indonesia (Tsai & Chen, 2017).”

# Peer review: Reviewer response for version 1

**DOI:** 10.5256/f1000research.138200.r155874

**Published:** 2022-11-17

**Authors:** Tita Alissa Bach

**Affiliations:** 1 Group Technology and Research, Life Sciences program, Healthcare Programme, Det Norske Veritas (DNV), Oslo, Norway

Abstract:

- This is maybe due to the word limit, but the abstract is not very clear. 

General feedback: 

- Are Covid 19 measures still valid in Indonesia? This study seems to be focusing on as if the measures were still ongoing. please adjust more timely to what is happening now and put your study in this context. I understand this study happened in the past when the measures were still valid, and this is fine, but the way reads now, it feels as if it was still like that, which is not true. Otherwise, your study does not really feel so relevant now when most countries have moved on from Covid 19 measures. Maybe put in the context as "lessons learned", and then generalise/extrapolate your findings and study into how people can learn from your findings *now* when the measures have been lifted. I believe that family resilience is crucial to cope with challenges, not necessarily only during covid measures. So maybe approach your study as a case study during covid measures. 
- In the discussion, I feel the unique characteristics of Indonesia are not really put into account. Families being resilient can be different in different countries due to local/cultural aspects. How is this for your findings in Indonesia?
- Why were participants only recruited from local universities? To my knowledge, married couples are more limited there. 
- It seems to be very ambitious to recruit the whole country. What was the reason not to target a specific study population? This is a survey study that can be done in collaboration with for example a maternity clinic, or wherever families/married couples hang around. In addition, targeting a study population will enable calculation of a response rate, which is lacking in your study. Once a response rate is available, non-response bias can be evaluated as this is a big factor for survey studies.
- You mention that you involved "participants from all around the archipelago" - yet only included several big cities. It is probably best to adjust these sentences? Also because in the same paragraph, you said "although we did not aim for a nationwide generalizability..." then why mention in the first place that you recruited the whole nation? 
- In which language was the survey for participants? Was the original survey (presumably in English) translated to Indonesian? How was the process? How was the Indonesian version validated? 
- Although it is negative findings based on Table 3, please discuss why demographic information does not influence. 
- Have you considered cultural differences as a factor here? Bandung and Jakarta may be rather similar, but when you start to involve Makassar and Ambon that can have different cultural attributes, this can explain some of your findings. Indonesia is too big to assume coherent and similar cultural factors and beliefs.
- Why only include typical demo info? What can make up resilience apart from included demo info? Could it be how long the couples have been married, whether both parents work, whether parents live together or live with other family members, access to support important for family resilience?
- Is the survey available for free for replication of your study?
- If the survey is online, do you have data for the average time someone took to finish the survey? You wrote that it takes about 15-20 minutes, is this the case though for your participants? 

Individual characteristics:

- "number of children" not child.

Is the work clearly and accurately presented and does it cite the current literature?

Yes

If applicable, is the statistical analysis and its interpretation appropriate?

I cannot comment. A qualified statistician is required.

Are all the source data underlying the results available to ensure full reproducibility?

Partly

Is the study design appropriate and is the work technically sound?

Yes

Are the conclusions drawn adequately supported by the results?

Yes

Are sufficient details of methods and analysis provided to allow replication by others?

Partly

Reviewer Expertise:

healthcare, psychology, human factors, sociology, behavioural and social sciences

I confirm that I have read this submission and believe that I have an appropriate level of expertise to confirm that it is of an acceptable scientific standard, however I have significant reservations, as outlined above.

# Response

**Published:** 2022-12-07

**Authors:** Tery Setiawan

**Affiliations:** Radboud University Nijmegen, The Netherlands

We would like to truly thank the reviewer for very insightful remarks and suggestions for the manuscript. We believe that your feedback has helped the manuscript greatly improve. The followings are responses to your remark/suggestion:

 Response to Reviewer 1 Comments

 Abstract:

 This is maybe due to the word limit, but the abstract is not very clear.

**Response**: Thank you for your feedback on our abstract. We have added more information to revise the abstract accordingly.

 General feedback:

 Are Covid 19 measures still valid in Indonesia? This study seems to be focusing on as if the measures were still ongoing. please adjust more timely to what is happening now and put your study in this context. I understand this study happened in the past when the measures were still valid, and this is fine, but the way reads now, it feels as if it was still like that, which is not true. Otherwise, your study does not really feel so relevant now when most countries have moved on from Covid 19 measures. Maybe put in the context as "lessons learned", and then generalise/extrapolate your findings and study into how people can learn from your findings now when the measures have been lifted. I believe that family resilience is crucial to cope with challenges, not necessarily only during covid measures. So maybe approach your study as a case study during covid measures.

**Response**: Thank you for your suggestion on extrappolating the current findings. We agree that, indeed, the findings can be generalized to different circumstances. For that, we have revised it accordingly.

 The revision is incorporated in the following part (of the last paragraph of the text) : “Finally, the current findings suggest that unexpected challenge or a sudden change is inevitably part of every family life. One way to overcome this is by adapting family resilience as a system, in which the adaptation is largely dependent on the way family members utilize their relationship and exchange of social support (Masten, 2019).”

 In the discussion, I feel the unique characteristics of Indonesia are not really put into account. Families being resilient can be different in different countries due to local/cultural aspects. How is this for your findings in Indonesia?

**Response**: Thank you for your comment on the unique characteristics of Indonesian families. We agree with this and we have responded to the comment accordingly in the text.

 The revision is incorporated in the following part: “In addition, we should also take into account by which family systems work in Indonesia. Reciprocation between parents and children are still regarded highly in the Asian context, including Indonesia (Tsai & Chen, 2017).”

 Why were participants only recruited from local universities? To my knowledge, married couples are more limited there.

**Response**: Thank you for your comment on this. We agree that we should have been more clear on the reason of recruiting from local universities. We have revised it accordingly in the text.

 The revision is incorporated in the following part: “Employing networks of local universities enable us to reach alumni and their extended networks.”

 It seems to be very ambitious to recruit the whole country. What was the reason not to target a specific study population? This is a survey study that can be done in collaboration with for example a maternity clinic, or wherever families/married couples hang around. In addition, targeting a study population will enable calculation of a response rate, which is lacking in your study. Once a response rate is available, non-response bias can be evaluated as this is a big factor for survey studies.

**Response**: Thank you for your comment on the data collection procedure. We agree that having a cooperation with certain institutions would enable us gather a specific set of sample and thus, enabling us to calculate a sampling representativeness. However, the aim of the study itself was to capture FQOL from various areas where we were able to access in such a short time. Therefore, this was the best solution available at the time.

 You mention that you involved "participants from all around the archipelago" - yet only included several big cities. It is probably best to adjust these sentences? Also because in the same paragraph, you said "although we did not aim for a nationwide generalizability..." then why mention in the first place that you recruited the whole nation?

**Response**: Thank you for your correction. We completely agree to revise such statements.

 The revision is incorporated in the following part: “This study was conducted online from August 2021 until October 2021 using Qualtrics covering a wide coverage of Indonesia”.

 In which language was the survey for participants? Was the original survey (presumably in English) translated to Indonesian? How was the process? How was the Indonesian version validated?

**Response**: Thank you for your comment on the process of the translation. We agree that we should have made it more explicit in the text. Therefore, we have revised the part accordingly.

 The revision is incorporated in the following part: “To serve the purpose of this study, we translated the measures into Indonesian language. Specifically for parental stress and family resilience measures, we checked our translation with the Indonesian studies that have involved the measures previously...”

 Although it is negative findings based on Table 3, please discuss why demographic information does not influence.

**Response**: We have added a plausible explanation in response to this. 

 The revision is incorporated in the following part: “It is also worth discussing why demographic characteristics are not related to the FQOL. First, by the time the survey was underway, most family members, irrespective of their gender, education and a number of children, were experiencing a similar change in their quality of life. After all, the Covid-19 pandemic has been shown to affect most, if not all, families in Indonesia (SMERU et al., 2021)…”

 Have you considered cultural differences as a factor here? Bandung and Jakarta may be rather similar, but when you start to involve Makassar and Ambon that can have different cultural attributes, this can explain some of your findings. Indonesia is too big to assume coherent and similar cultural factors and beliefs.

**Response**: Thank you for your remark on the cultural differences. We acknowledge that local cultural differences might have played a role in determining our findings. However, we do not have sufficient information to infer such arguments. Although we have incorporated your remark on this in our study limitation in a discussion section.

 The revision is incorporated in the following part: “One, we realize that our small sample may not reflect the whole picture of the family situation across Indonesia and therefore, we cannot infer anything about local cultural differences across the regions These differences, of course, might have played a role in determining the level of FQOL.”   

 Why only include typical demo info? What can make up resilience apart from included demo info? Could it be how long the couples have been married, whether both parents work, whether parents live together or live with other family members, access to support important for family resilience?

**Response**: Thank you for your comment and we completely agree with this. In conducting this study, we were warned of the ‘online exhaustion’ among people during the Covid-19 pandemic. Therefore, we had to sacrifice some of the important questions, such as the ones you mentioned, to ensure that the completion rate is quite enough for us to compute the main analyses. Nevertheless, we have also incorporated this information in our study limitation to encourage others scholars to include them in future studies.

 The revision is incorporated in the following part: “The anticipation of ‘online exhaustion’ also encouraged us to reduce a number of questions, such as years of marriage and co-residing with parent(s), so that we would still have a high completion rate.”  

 Is the survey available for free for replication of your study?

**Response**: Thank you for your question. Yes, the survey, along with its protocol, is available online so that other scholars are able to replicate the study.

 The statement is incorporated in the following part: “The protocol, along with the measures employed in this study, can be used for replication and future studies.”

 If the survey is online, do you have data for the average time someone took to finish the survey? You wrote that it takes about 15-20 minutes, is this the case though for your participants?

**Response**: Thank you for your valuable question on the completion time. We have responded to the question accordingly in the text.

 The revision is incorporated in the following part: “In the end, we did not compute the time average taken by participants, because we set the online survey to allow participants to take a break during their participation thus, allowing multiple sessions or even days to finish the full questionnaire.”

 Individual characteristics:

 "number of children" not child.

**Response**: Thank you for your correction. We have revised it accordingly.
